# Le syndrome du canal carpien chez les patients hémodialysés chroniques

**DOI:** 10.11604/pamj.2013.14.19.387

**Published:** 2013-01-14

**Authors:** Houda Mbarki, Amine Akrichi, Amine Lazrak, Chakib Maaroufi, Aouatif El Midaoui, Nabil Tachfouti, Wafa Jellouli, Mohamed Arrayhani, Mohamed Faouzi Belahsen, Tarik Sqalli

**Affiliations:** 1 Service de néphrologie, CHU Hassan II, Fès, Maroc; 2 Service de neurologie, CHU Hassan II, Fès, Maroc; 3 Laboratoire d’épidémiologie, de recherche clinique et de santé communautaire, Faculté de Médecine et de Pharmacie de Fès, Maroc

**Keywords:** Béta2-microglobuline, hémodialyse chronique, syndrome du canal carpien, electromyogramme, Beta2-microglobulin, chronic hemodialysis, carpal tunnel syndrome, EMG

## Abstract

Le syndrome du canal carpien (SCC) regroupe l'ensemble des signes et symptômes liés à la compression du nerf médian dans le canal carpien. Cette manifestation de l'amylose à béta2-microglobuline est une complication fréquente de l'hémodialyse au long cours. L'objectif de ce travail est d'analyser les caractéristiques du SCC et de déterminer les facteurs liés à sa survenue chez les hémodialysés chroniques. Nous rapportons une étude transversale monocentrique, menée au 3ème trimestre de l'année 2009, portant sur les patients adultes hémodialysés chroniques au service de Néphrologie-Hémodialyse au CHU de Fès. 59 patients ont accepté de participer à l'étude. Leurs âge moyen est de 48 ± 15 ans avec un sex-ratio de 0,9. Ils bénéficient tous de l'hémodialyse intermittente à raison de 10 à 12 heures par semaine, par une membrane de dialyse en polysulfone à basse perméabilité. La durée moyenne en hémodialyse est de 83 ± 6,5 mois. La prévalence du SCC dans notre centre est de 30,5%. L'électromyogramme (EMG) a confirmé la suspicion clinique du SCC chez 11 patients et a diagnostiqué un SCC chez 8 patients asymptomatiques. La comparaison statistique entre les deux groupes de patients avec et sans SCC a démontré que la survenue de ce syndrome est liée à: l'âge actuel, l'âge avancé à la mise en hémodialyse, le sexe féminin, l'excès pondéral, et l'abord vasculaire. Le SCC est une complication fréquente de l'hémodialyse chronique. L'amélioration de la qualité de dialyse permettrait de réduire le risque de survenue du SCC.

## Introduction

La survie prolongée des insuffisants rénaux, en partie liée à l'amé1ioration des techniques de suppléance extra-rénales, fait apparaitre chez ces patients une pathologie de l'appareil locomoteur à l'origine d'une morbidité importante, en particulier en cas d'hémodialyse au long cours. Le syndrome du canal carpien (SCC) regroupe l'ensemble des signes et symptômes liés à la souffrance du nerf médian au niveau du canal carpien. Il fut mis en évidence, en tant que complication de l'hémodialyse, en 1975 par Waren et Otieno [1]. L'existence de dépôts d'une substance amyloïde au niveau de la synoviale des tendons fléchisseurs au canal carpien fut rapportée pour la première fois en 1980 par Assenat et al [2]. Un type particulier d'amylose secondaire, constituée par des polymères de béta2-microglobuline (β-2 m) fut identifié comme le constituant de ces dépôts chez les patients hémodialysés chroniques, en 1985 par Gejyo [3]. Ces dépôts amyloïdes furent aussi documentés chez les hémodialysés, à l'origine d'arthropathies érosives, de doigts à ressaut et de ruptures tendineuses [4–6]. A l'heure actuelle, les progrès réalisés en hémodialyse avec les membranes biocompatibles, l'hémodialyse à haute perméabilité, l'amélioration de la qualité de l'eau etc’ ont permis de réduire la prévalence du SCC dans les centres d'hémodialyse. Malgré ces avancées, des cas de SCC subsistent dans nos centres. L'objectif de cette étude est d'analyser les caractéristiques du SCC et les facteurs liés à sa survenue chez les patients hémodialysés chroniques dans notre centre.

## Méthodes

**Type d’étude**: Il s'agit d'une étude transversale monocentrique, menée au troisième trimestre de l'année 2009. Elle a porté sur les patients hémodialysés chroniques du service de néphrologie-hémodialyse du CHU de Fès.

**Patients**: Ont été inclus tous les patients adultes hémodialysés chroniques depuis au moins un an qui ont accepté de participer à l'étude.

**Eléments recueillis**: Les données sociodémographiques ont été recueillies à partir des dossiers médicaux des patients. La présence de douleur ou de paresthésie dans le territoire du nerf médian a été recherchée à l'interrogatoire. Deux manœuvres cliniques ont été réalisés aux deux mains et considérées comme positives si elles reproduisaient la douleur ou les paresthésies spontanées dans le pouce, l'index et le médius (territoire du nerf médian). Il s'agit des tests de Tinel et de Phalen.

Les examens biologiques suivants ont été relevés chez tous les patients avant la séance d'hémodialyse: C-réactive protéine (CRP), béta2-microglobuline, parathormone intacte (PTHi 1–84). Nous avons défini l'inflammation par un taux de CRP >6mg/l et l'hyperparathyroidie par un taux de PTHi >9 fois la limite supérieure de la normale.

Le bilan électromyographique (EMG) a été réalisé chez tous les patients par le même médecin neurologue, dans la même pièce et les mêmes conditions de température. Nous avons considéré l'EMG comme le gold standard pour le diagnostic du SCC que nous avons défini par: l'allongement des latences distales motrices du nerf médian; la diminution de l'amplitude de la réponse sensitive du nerf médian; et la diminution de la conduction sensitive du nerf médian.

### Description des tests utilisés

Test de Tinel: l'examinateur applique un coup sur le pouce à l'endroit où passe le nerf médian; une petite sensation de décharge électrique ressentie dans un ou plusieurs doigts peut indiquer un dommage du nerf médian.

Test de Phalen: le patient, poignet fléchi, met ses mains dos contre dos en poussant pendant une minute. La présence du SCC peut être évoquée si un ou plusieurs symptômes, tels qu'une sensation de décharge électrique ou d'engourdissement, sont ressentis dans les doigts pendant la minute en question.

EMG: le neurologue place de fines électrodes sur le trajet du nerf médian, envoie de faibles décharges électriques et mesure la vitesse et l'amplitude de la conduction sensitive dans le nerf médian sur deux segments doigt-poignet et la latence motrice distale du nerf médian entre le poignet et le thénar.

**Qualité de l'eau:** La qualité de l'eau traitée utilisée pour l'hémodialyse est contrôlée régulièrement par le « laboratoire d'analyses agro-alimentaires et d'environnement: Protège Maroc ». Elle est certifiée conforme aux normes chimiques et bactériologiques exigées par l'arrêté du ministère de la santé n° 808-02 du 25 hija 1423 fixant les normes techniques des centres d'hémodialyse et aux normes de la nomenclature européenne [7].

### Analyse statistique

Elle a été effectuée à l'aide du logiciel Epi-info version 2000. Les patients ont été répartis en deux groupes, selon la présence ou non du SCC à l'EMG. Le premier groupe comprend les patients chez qui un SCC est diagnostiqué. Le deuxième comprend ceux ne présentant pas le SCC. Une analyse univariée a ensuite été effectuée pour étudier l'association entre la présence ou non du SCC et un certain nombre de paramètres cliniques et paracliniques. Le test de Chi-2 a permis de comparer les variables qualitatives. Pour les variables quantitatives nous avons choisi le test de Student. Une valeur de p inférieure à 0,05 a été considérée comme significative. Le nombre de patients inclus dans l'étude n'a pas permis la réalisation d'analyse multivariée.

## Résultats

Parmi 84 patients hémodialysés dans l'unité d'hémodialyse du centre hospitalier universitaire Hassan II de Fès, 59 patients ont accepté de participer à l'étude. L'âge moyen est de 48 ± 15 ans avec un sex-ratio de 0,9. Touts nos patients sont droitiers. Ils bénéficient tous de l'hémodialyse chronique intermittente à raison de 10 à 12 heures par semaine par le même type de membrane de dialyse, il s'agit d'une membrane en polysulfone à basse perméabilité. La fistule artérioveineuse (FAV) est l'abord vasculaire utilisé chez tous les patients. La durée moyenne en hémodialyse est de 83 ± 6,5 mois, avec des extrêmes de 12 à 183 mois. Les caractéristiques sociodémographiques et clinico-biologiques des patients sont présentées dans le Tableau 1.

**Tableau 1:** Caractéristiques clinico-biologiques des patients hémodialysés chroniques au centre

Country	years	N	Age					Diagnosis (number)	
			Mean Age	Std. Deviation	Std. Error	Minimum	Maximum	Ochitis	Torsion
France	1987–1994	48	12.96	2.133	.308	9	17	0	48
Kenya	1981–1982	24	26.42	7.471	1.525	13	40	20	4
Nigeria	1983–1986	52	30.42	8.472	1.175	13	53	51	1
R.S.A	1997–2003	83	27.23	12.668	1.390	9	56	53	30
Rwanda	1995–1996	10	29.40	8.784	2.778	20	52	10	0
Uganda	2003–2011	15	24.87	8.158	2.106	13	40	11	4
Uganda-2	1980–1981	73	23.88	8.071	.945	12	37	50	23
**Total**		**305**	**24.62**	**10.548**	**.604**	**9**	**56**	**195**	**110**

Les signes fonctionnels relevés sont les paresthésies dans le territoire du nerf médian chez 25,4% des patients (15 cas); une douleur à type de brulure ou d’élancement chez 12% (7 cas). L'exacerbation de la symptomatologie au cours de la séance d'hémodialyse est rapportée par 8,5% (5 cas). L'examen clinique retrouve le signe de Phalen chez 8,5% (5 cas), le signe de Tinel chez 5% (3 cas), l'atrophie thénarienne chez 8,5% (5 cas). Aucun cas d'hypoesthésie n'est noté.

Nous avons diagnostiqué le SCC chez 18 patients sur 59 (30,5%). Ces patients se répartissent en 16 femmes et 2 hommes, âgés de 42 à 79 ans. L'anurie (diurèse <400 ml/jour) est retrouvée chez 17 (94%) d'entre eux. Leurs principales caractéristiques sont présentées dans le Tableau 2. Le SCC est unilatéral dans 3 cas et est bilatéral dans 15 (soit au total 33 SCC diagnostiqués). Des signes de sévérité à l'EMG sont objectivés dans 50% des SCC diagnostiqués.

L'EMG a confirmé la suspicion clinique du SCC chez 11 patients et a permis de diagnostiquer un SCC chez 7 patients cliniquement asymptomatiques. La sensibilité des signes fonctionnels pour le diagnostic du SCC est de 53%, leur spécificité est de 89,5%. Les man'uvres cliniques sont spécifiques pour le SCC (89% pour le test de Tinel et 99% pour le Phalen), mais elles sont peu sensibles (15% pour le Tinel et 18% pour le Phalen).

En analyse univariée, la comparaison entre les patients porteurs de SCC et ceux indemnes a démontré que les patients du 1er groupe sont actuellement plus âgés [8]. Dans notre série, on note une faible sensibilité des man'uvres cliniques. En effet, l'EMG a permis de dépister des lésions infra-cliniques. Cependant, malgré la bonne spécificité des tests cliniques dans notre étude, la réalisation d'un EMG de confirmation avant tout traitement chirurgical nous semble nécessaire.

**Tableau 2:** Caractéristiques clinico-biologiques des deux groupes de patients (avec et sans syndrome de canal carpien; analyse univariée)

Paramètres	SCC absent n = 41	SCC présent n = 18	P
Age	42,5 ± 13	59 ± 11	0,0001
Sexe féminin (%)	36,6	89	0,0001
Age à la mise en dialyse (ans)	36 ± 13	51 ± 12	0,0002
Durée en HD (mois)	77,6 ± 51	96,3 ± 68	0,19
Excès pondéral (%)	14.6	40.6	<0,04
Diurèse résiduelle <400 ml/jour (%)	50	94	0,01
β-2 m (mg/l)	56,6 ± 12	48 ± 15,9	0,04
Hyperparathyroidie (%)	65,9	66,7	NS
CRP (mg /l)	7,72 ± 4,2	13,6 ± 9	NS

La prévalence de 30,5% du SCC chez nos patients hémodialysés chroniques concorde avec la série tunisienne de Namazi et al. en 2007 (42,7%) [9], alors qu'elle est plus élevée en comparaison avec la série américaine de Gilbert et al. en 1988 (9%) [10], et celle Allemande de Schwalb et al. en 1996 (16,3%) [11]. Cette variabilité peut être expliquée par la différence de la durée de dialyse dans ces études, grâce à l'amélioration de la survie des patients au fil des années ainsi que par la disparité des critères (cliniques, élecrophysiologiques, ou histologiques) considérés pour le diagnostic du SCC dans les séries.

La durée prolongée en hémodialyse est retrouvée comme facteurs de risque de survenue du SCC dans la littérature, alors qu'elle est statistiquement non significative dans notre série [6, 12–15]. Ce fait pourrait être expliqué par le nombre faible de nos patients. Notre étude confirme le fait que le risque de survenue du SCC augmente avec l'âge. L'âge avancé à la mise en hémodialyse est également lié à la survenue du SCC dans la littérature [6, 9, 10]. Jadoul et al. [16] incriminent l'altération qualitative et/ou quantitative liée à l'âge des glyosaminoglycanes rentrant dans la formation des dépôts amyloïdes. La prédominance féminine du SCC chez les patients hémodialysés chroniques rapportée par la littérature est retrouvée dans notre série [4, 10, 17–21]. Elle est également rapportée par les auteurs chez les patients non dialysés porteurs de SCC [22, 23] (Tableau 3).

**Tableau 3:** Facteurs associés à la survenue du syndrome de canal carpien chez les patients hémodialysés dans la littérature

Paramètres	Notre série n = 18 sur 59	Schwalbe et al. [11] n = 7 sur 43	Gilbert et al. [10] n = 44 sur 485
Age (moyenne)	59 ± 11*	51 ± 13*	58 (33–74) *
Sexe féminin (%)	88,9*	25,6*	66,7*
Age à la mise en dialyse (moyenne)	51*	–	49,8 (23–72) *
Durée en HD (mois)	96,3	71 ± 50*	98,4 (3–180) *

* p < 0, 05

L'excès pondéral est également lié à la survenue du SCC chez nos patients. Cette relation n'a pas été retrouvée dans l'étude de Schwalbe et al. [11]. La survenue du SCC est significativement associée à l'anurie dans notre étude. Ceci peut être expliqué par un défaut d’élimination rénale de la ‘- 2 m chez les hémodialysés. Les auteurs [24] ont démontré que les concentrations les plus élevées de la ‘- 2 m sont retrouvées chez les patients urémiques oligo-anuriques.

L'étiopathogénie de la souffrance du nerf médian dans le canal carpien chez les patients hémodialysés chroniques est multifactorielle. L'infiltration de la synoviale du tendon fléchisseur par des dépôts amyloïdes semble être l'élément clé de la genèse du SCC [25]. Au cours de l'insuffisance rénale chronique, la dégradation de la filtration glomérulaire et de la réabsorption tubulaire s'accompagnent d'une augmentation progressive du taux sanguin circulant de la β-2 m [24]. Cette dernière étant une molécule de moyen poids moléculaire est mal épurée en hémodialyse intermittente. L'augmentation de sa concentration sérique a été proposée à l'origine des dépôts amyloïdes à β-2 m chez le patient urémique [24, 26].

Le taux sérique moyen circulant de la β-2 m retrouvé dans notre série est paradoxalement plus élevé chez les patients non porteurs de SCC. Toutefois, le taux de β-2 m dans les deux groupes reste supérieur à celui rapporté dans la littérature témoignant d'une mauvaise épuration des moyennes molécules [11]. Le Kt/V de l'urée, marqueur de la qualité d’épuration des molécules de faible poids moléculaire, n'a pas été étudié dans ce travail.

Le type de dialyse joue aussi un rô1e important dans l'accumulation de la β-2 m [11]. En effet, les membranes cellulosiques (cuprophane) ont été incriminées du fait de leur plus faible capacité d’épuration par rapport aux membranes synthétiques [27, 28]. Toutefois, des cas de dépôts amyloïdes ont été décrits, mais moins fréquemment chez des hémodialysés utilisant des membranes synthétiques et même en cas de dialyse péritonéale [29–32]. Plusieurs auteurs spéculent sur le rôle d'autres facteurs dans l'accumulation de la β-2 m et dans la genèse des dépôts amyloïdes à β-2 m en hémodialyse chronique, notamment ceux liés à la biocompatibilité des membranes de dialyse ainsi qu’à la qualité de l'eau pour hémodialyse et donc du dialysat. Une baisse de la libération des médiateurs de l'inflammation, des protéases et des radicaux libres oxygénés observée avec les membranes plus biocompatibles est proposée pour argumenter cette hypothèse [11, 32]. Enfin, l'utilisation de membranes à haute perméabilité protège contre les dépôts de β-2 m et diminue le risque de SCC [32].

Dans notre étude, l'élévation de la CRP est retrouvée chez les 2 groupes de patients témoignant de l'inflammation chronique. Cependant, elle n'est pas statistiquement liée à la présence du SCC. Des études ont démontré que la micro-inflammation chez les malades urémiques est associée à une augmentation de la morbidité globale [33]. Le dosage de la micro-CRP chez nos patients n'a pas été effectué.

Les facteurs vasculaires semblent aussi jouer un rôle important. Il s'agit de séquelles de modifications de l'hémodynamique dues à la présence de la FAV. Cette dernière entrainerait une hyperpression nerveuse dans la main qui serait cause de l'‘dème, qui lui-même occasionnerait secondairement la compression du nerf médian dans le canal carpien [10, 17]. D'autres auteurs suggèrent que les symptômes du SCC sont causés par l'ischémie due au vol vasculaire provoqué par la FAV. Mes ces facteurs vasculaires ne permettent pas d'expliquer les cas de patients porteurs d'une FAV unilatérale pour lesquels le syndrome du canal carpien est bilatéral [10, 22, 23].

La transplantation rénale est le seul traitement actuellement capable de stopper la progression de l'amylose à β-2 m. La préservation de la fonction rénale résiduelle et la correction de l'excès pondéral permettront peut être une diminution de la prévalence du SCC. En dehors de la transplantation, d'autres facteurs comme l'utilisation des membranes à haute perméabilité, et les membranes biocompatibles ainsi que l'utilisation du dialysat ultrapure peuvent aussi conduire à une baisse de la prévalence de la maladie. Un taux de β-2 m inférieur à 25 mg/l chez les hémodialysés chroniques témoignant d'une bonne épuration des moyennes molécules doit être exigé. Ces mesures peuvent apporter un grand bénéfice surtout dans les pays ou la transplantation rénale n'est pas disponible et pour les patients ne pouvant pas en bénéficier [32, 34, 35].

## Conclusion

La prévalence du SCC dans notre centre est de 30,5%. Nous avons trouvé une difficulté à la comparer avec la littérature vue la grande variabilité des modalités d'hémodialyse d'un centre à l'autre. Cette étude nous a permis de préciser les particularités cliniques et étiopathogéniques conférées au SCC par l'atteinte rénale. Son caractère transversal n'a pas permis par ailleurs de déterminer les facteurs de risque de survenue du SCC. Une étude de cohorte ou cas-témoin portant sur un plus grand nombre de patients serait nécessaire pour vérifier nos résultats.
